# Shorter telomere length increases age‐related tumor risks in von Hippel‐Lindau disease patients

**DOI:** 10.1002/cam4.1134

**Published:** 2017-08-04

**Authors:** Jiang‐Yi Wang, Shuang‐He Peng, Xiang‐Hui Ning, Teng Li, Sheng‐Jie Liu, Jia‐Yuan Liu, Bao‐An Hong, Nie‐Nie Qi, Xiang Peng, Bo‐Wen Zhou, Jiu‐Feng Zhang, Lin Cai, Kan Gong

**Affiliations:** ^1^ Department of Urology Peking University First Hospital Beijing China; ^2^ Institute of Urology Peking University Beijing China; ^3^ National Urological Cancer Center Beijing China

**Keywords:** Cancer syndrome, phenotypic variability, telomere length, tumor risk, von Hippel‐Lindau disease

## Abstract

Von Hippel‐Lindau (VHL) disease is a rare autosomal dominant cancer syndrome caused by alterations of *VHL* gene. Patients are predisposed to develop pheochromocytomas and solid or cystic tumors of the central nervous system, kidney, pancreas, and retina. Remarkable phenotypic heterogeneity exits in organ involvement and tumor onset age between and within VHL families. However, no reliable markers have been found to predict the age‐related tumor risks in VHL patients. A large Chinese cohort composed of 300 VHL patients and 92 healthy family controls was enrolled in our study. Blood relative telomere length was measured in 184 patients and all the controls available for genomic DNA samples. Age‐related risks for the five major VHL‐associated tumors were evaluated using Kaplan–Meier plots and Cox regression analysis. Differences in clinical phenotype were observed between Chinese cohort and the United Kingdom cohort. VHL patients showed significantly shorter telomere length than healthy family controls(*P* = 0.0183), and a positive correlation was found between telomere length and onset age of the five major tumors, respectively. Moreover, patients in the shorter telomere group (age‐adjusted telomere length ≤ 0.44) suffered higher age‐related risks for VHL‐associated central nervous system hemangioblastomas (HR: 1.879, *P* = 0.004), renal cell carcinoma (HR: 2.126, *P* = 0.002) and pancreatic cyst and neuroendocrine tumors (HR: 2.093, *P* = 0.001). These results indicate that blood shorter telomere length is a new biomarker for age‐related tumor risks in VHL patients, which will be crucial to genetic counseling and future research about the role of telomere shortening in the pathogenesis of VHL‐associated tumors.

## Introduction

Von Hippel‐Lindau (VHL) disease (MIM 193300) is a rare autosomal dominant cancer syndrome caused by germline mutations in the *VHL* gene 1, 2. It is characterized by various early‐onset tumors, including central nervous system hemangioblastomas (CHB), retinal hemangioblastomas, clear cell renal cell carcinoma (RCC), pancreatic cyst and neuroendocrine tumors (PCT), pheochromocytomas (PHEO), endolymphatic sac tumors (ELST), epididymal and broad ligament cystadenomas 2, 3, 4, 5. The birth incidence of VHL disease is about 1 in 36000–53000 across the world, with a high penetrance of more than 90% by 70 years old 6, 7, 8, 9. Patients may be affected by cancers from childhood and throughout their lifetime.

There is an obvious phenotypic heterogeneity in the tumor types and onset age between and within VHL families. Clinically, VHL disease has been classified into two types depending on patients' predisposition to pheochromocytoma. Genotype–phenotype correlations have been well constructed: Type 1 patients usually harbor truncating mutations and confer a lower risk for pheochromocytomas, while Type 2 patients are characterized by missense mutations and with an increased risk for pheochromocytomas 8, 10, 11, 12, 13. In recent studies, researchers find that large *VHL* gene deletions involving the adjacent gene *C3orf10* (*BRK1*) are associated with a definite low risk of RCC 7, 14, 15, 16. Although the genotype–phenotype correlations partially explain the variation in tumor types, it has limited clinical utility because quite a part of the patients don't conform to the rule and families may move from one type to another with time 7. In addition, no markers have been found to correlate to tumor onset age variability and age‐related tumor risks in VHL patients. Our previous study demonstrated that telomere shortening was contributable to the genetic anticipation in successive generations of Chinese VHL families, indicating that telomere length might be a potential biomarker for the phenotypic variability 17.

Telomeres are repetitive nucleotide sequences that protect chromosomes from degradation, fusion and undue recombination, and become shorter during each cell division 18, 19. Critically short telomeres lead to genetic instability and increase risks of age‐related disease including cancers 20, 21. Peripheral blood leukocyte telomere length has been reported to be a potential biomarker of tumor risk for various types of familiar and sporadic tumors, including hereditary non‐polyposis colorectal cancer(Lynch syndrome), hereditary prostate cancer, familial and sporadic ovarian cancer, renal cell carcinoma and pancreatic cancer 22, 23, 24, 25, 26, 27. VHL disease seems similar to some hereditary disease such as Lynch syndrome, and shorter telomere length contributes to the earlier onset age in the next generations than their parental generations within VHL families. However, no study has been carried out on the relationship between blood telomere length and age‐related tumor risks in VHL patients.

Given the relevance of telomeres and their function in tumor, we hypothesized that shorter blood telomere length is a new biomarker for age‐related tumor risks in VHL patients. The validation of this assumption will lead to a notion that telomere shortening may participate in the pathogenesis of VHL‐associated tumors, and telomere measurement will provide valuable information for genetic counseling. Herein, we tested this assumption in a cohort of Chinese VHL patients.

## Patients and Methods

### Ethics Statement

This study was approved by the Medical Ethics Committee of Peking University First Hospital (Beijing, China) and written informed consent was obtained from all subjects.

### Patients and samples

From 2009 to 2016, 348 patients from 133 families were diagnosed with VHL disease at the Peking University First Hospital based on the clinical criteria and *VHL* gene detection as previously described 28. The *VHL* mutation analysis was conducted by direct sequencing and multiplex ligation‐dependent probe amplification (MLPA) P16‐C2 kits (MRC‐Holland, Amsterdam, Netherlands). Clinical data was collected on all tumor‐affected individuals and asymptomatic *VHL* mutation carriers. In these families, 48 patients were excluded because of obscure clinical information. Therefore, a total of 300 patients from 120 families were enrolled in this study. Clinical records and reports were reviewed to determine the onset age of the five major VHL lesions. In all the people enrolled, 184 patients and 92 healthy family members with available peripheral blood DNA samples were brought into analysis for the relationship between relative telomere length (RTL) and age‐related tumor risks.

A published cohort comprised of 573 VHL patients from 200 kindreds in United Kingdom was included to analyze the difference between VHL patients in China and UK 8. The genetic and clinical data was downloaded, and 525 patients were available for accurate genotypic information.

### Relative telomere length measurement

Genomic DNA was extracted from peripheral blood leukocyte by using a blood DNA extraction kit (Tiangene). RTL was quantified by measuring copy number ratio of telomere repeats (T) to the single copy gene 36B4 (S) using qRT‐PCR described by Cawthon 29. The PCR reaction was run in an ABI 7500 PCR instrument, containing 5 *μ*L 2 × SYBR master mix (Takara), 30 ng genomic DNA, 300 nmol/L telomere primer Tel1 (5^′^‐GGTTTTTGAGGGTGAGGGTGAGGGTGAGGGTGAGGGT) and 900 nmol/L telomere primer Tel2 (5^′^‐TCCCGACTATCCCTATCCCTATCCCTATCCCTATCCCTA), or 200 nmol/L single copy gene primer 36B4u (5^′^‐CAGCAAGTGGGAAGGTGTAATCC) and 500 nmol/L 36B4d (5^′^‐CCCATTCTATCATCAACGGGTACAA) 17. Whenever possible, samples from different groups were run in the same plate. The running profile is of 95°C for 30 sec and 40 cycles of 95°C for 15 sec, 54°C for 2 min, and 72°C for 15 sec. A standard curve was constructed to assess the amplification efficiency (E) using a control DNA sample (male, 45 years old) diluted by 1/4 serial from 50 ng to 0.19 ng, and the same sample was detected in every batch of PCRs as the inter‐run calibration. The telomere repeats (T) was described by (E_Tel, sample_)^−Ct (Tel, sample)^/(E_Tel, calibrator_)^−Ct (Tel, calibrator)^, and the copy number of 36B4 (S) was (E_36B4, sample_)^−Ct (36B4, sample)^/(E_(36B4, calibrator_)^−Ct (36B4, calibrator)^. RTL was calculated by T/S. Age‐adjusted relative telomere length (aRTL) was obtained based on the telomere‐age curve constructed in our previous study.

### Statistical analysis

Mann‐Whitney test and *t* test were used to evaluate the difference of aRTL and mean onset age of VHL‐associated tumors between groups, respectively. Chi‐square test was used to compare the differences in frequencies of tumors and mutation type distribution between Chinese and UK VHL patients. The correlation between aRTL and tumor onset age was described with Spearman's rank correlation coefficient.

We used Kaplan–Meier plots and log‐rank analysis to describe the distribution of onset age of different tumor types. For VHL‐associated tumor risks analysis, using Cox regression method, VHL patients were considered to be informative from birth until first diagnostic, last contact, or death. The development of the five major VHL related tumors were considered to be the end point.

Statistical analysis was performed using SPSS20.0, and *P* < 0.05 was considered to be statistically significant.

## Results

### Differences in genotypic and clinical characteristics of VHL patients between Chinese cohort and United Kingdom cohort

The diagnosis age and frequency of major VHL‐related tumors of Chinese cohort and United Kingdom cohort were displayed in Table 1. The mean age at diagnosis of the first lesion in Chinese VHL patients was 30.9 years old, which was 6.2 years older than that in UK cohort 8. Overall frequencies of CHB, PHEO and RA in this study were significantly lower than UK (CHB: 63.1% vs. 82%, *P* < 0.001, PHEO: 13% vs. 20%, *P* = 0.009, RA: 22.3% vs. 73%, *P* < 0.001), while the frequency of RCC was surprisingly higher (42.7% vs. 35%, *P* = 0.028). This suggested that the phenotypic variability in VHL disease also existed between different ethnic groups.

**Table 1:** Clinical characteristics of VHL patients in China and UK

Tumor	China (*n* = 300)			UK (*n* = 573)^a^			*P* c
	Affected *N* (%)	Not affected *N* (%)	Onset age (y) (Mean±SD)	Affected *N* (%)	Not affected *N*(%)	Onset age (y) (Mean)	
CHB	184 (61.3)	116 (38.7)	31.3 ± 11.9	470 (82)	103 (18)	30.9	<0.001
RCC	128 (42.7)	172 (57.3)	40.2 ± 11.2	201 (35)	372 (65)	39.7	0.028
PCT	140 (46.7)	160 (53.3)	36.1 ± 11.4	‐b	‐b	‐b	‐b
PHEO	39 (13.0)	261 (87.0)	33.7 ± 13.1	115 (20)	458 (80)	24.0	0.009
RA	67 (22.3)	233 (77.7)	28.5 ± 12.6	418 (73)	155 (27)	25.0	<0.001

CHB, central nervous system hemangioblastoma; RA, retinal angioma; RCC, renal cell carcinoma; PCT, pancreatic cyst or pancreatic tumor; PHEO, pheochromocytoma.

a Ong et al 8.

b Not described in the UK cohort.

c *P* value for the difference of overall penetrance in VHL patients between China and UK.

To evaluate whether the phenotypic variability between patients in China and UK was caused by differences in genotype. We compared the genotypic characteristics between China cohort and UK cohort (Table 2). The proportion of missense mutation in Chinese VHL patients was higher than UK (50.7% vs. 39.4%), while the proportion of frameshift/nonsense mutation was lower (21% vs. 32.8%). According to the classic genotype–phenotype correlation 8, patients with missense mutation had higher risk for pheochromocytomas. However, the overall frequency of pheochromocytomas in our study was significantly lower than UK. This gave us a clue that some other factors might lead to the phenotypic heterogeneity between VHL patients in China and UK.

**Table 2:** Genotypic characteristics of VHL patients in China and UK

Mutation type	China cohort	UK cohort	*P*
	Number of patients (%)	Number of patients (%)	
Large deletion	65 (21.7)	113 (21.5)	0.005
Frameshift/nonsense	63 (21.0)	172 (32.8)	
Splicing	12 (4.0)	18 (3.4)	
In‐frame deletion/insertion	8 (2.7)	15 (2.9)	
Missense	152 (50.7)	207 (39.4)	
Total	300 (100)	525 (100)	

### VHL patients showed shorter telomere length than healthy family controls

**Figure 1:**
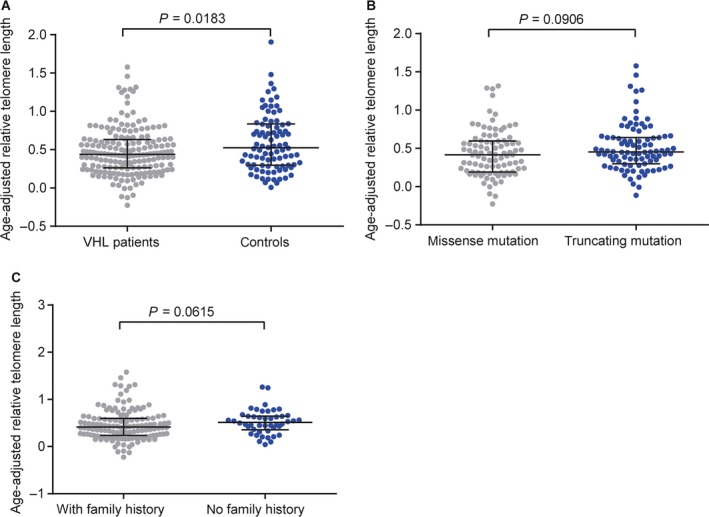
Age‐adjusted relative telomere length in VHL patients and healthy family controls. (A) Difference of age‐adjusted RTL in the VHL patients and healthy family controls. (B) Age‐adjusted RTL in missense mutation and truncating mutation subgroups. (C) Age‐adjusted RTL in VHL patients with and without family history.

To evaluate the difference of RTL between VHL patients and normal controls, we measured the RTL in 184 VHL patients and 92 healthy family members. As telomere is attrited with age, the RTL was adjusted by age according to the linear regression equation conducted in our previous study 17. As expected, VHL patients showed significantly shorter telomere length than healthy family controls(*P* = 0.018) (Fig. 1A), implying that VHL patients had abnormal telomere dynamics. No significant differences were observed between different mutation types and family history status(Fig. 1B–C).

### Positive correlation exists between relative telomere length and the onset age in tumor‐affected VHL patients

**Figure 2:**
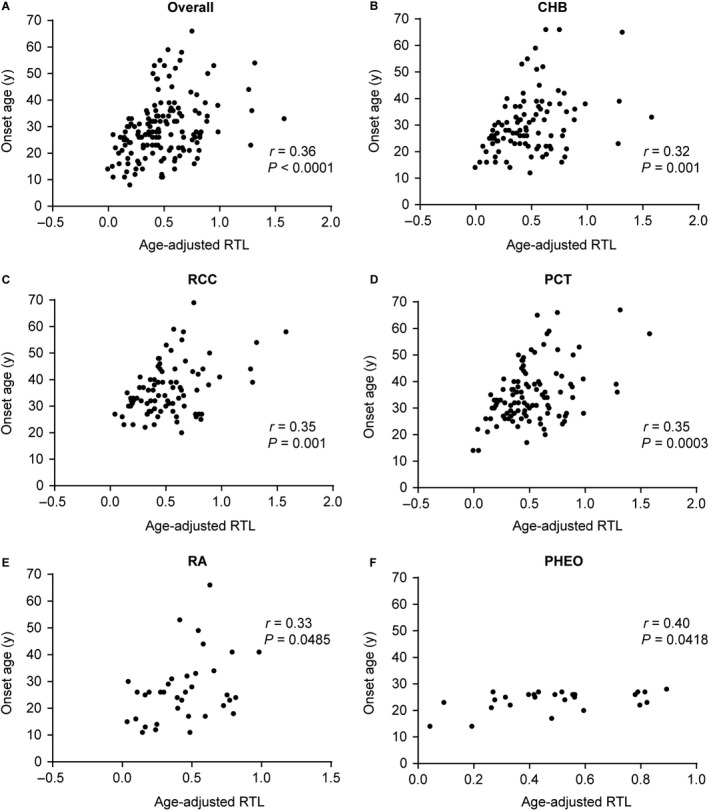
Correlations between age‐adjusted RTL and tumor onset age. Spearman's rank correlation coefficient was used to evaluate the relationship between age‐adjusted RTL and the onset age of the overall tumors(A), CHB(B), RCC(C), PCT(D), RA(E) and PHEO(F).

We next investigated whether aRTL was correlated to the onset age of VHL‐associated tumors in tumor‐affected patients. A positive correlation was found between aRTL and onset age of the first lesion in VHL disease (*r* = 0.36, *P* < 0.0001) (Fig. 2A). We further assessed whether aRTL was associated with the onset age of five VHL‐related tumors, respectively. Similar results were observed in CHB (*r* = 0.32, *P* = 0.001), RCC (*r* = 0.35, *P* = 0.001), PCT (*r* = 0.35, *P* = 0.0003), RA (*r* = 0.33, *P* = 0.0485) and PHEO (*r* = 0.40, *P* = 0.0418) subgroups (Fig. 2B–F).

**Figure 3:**
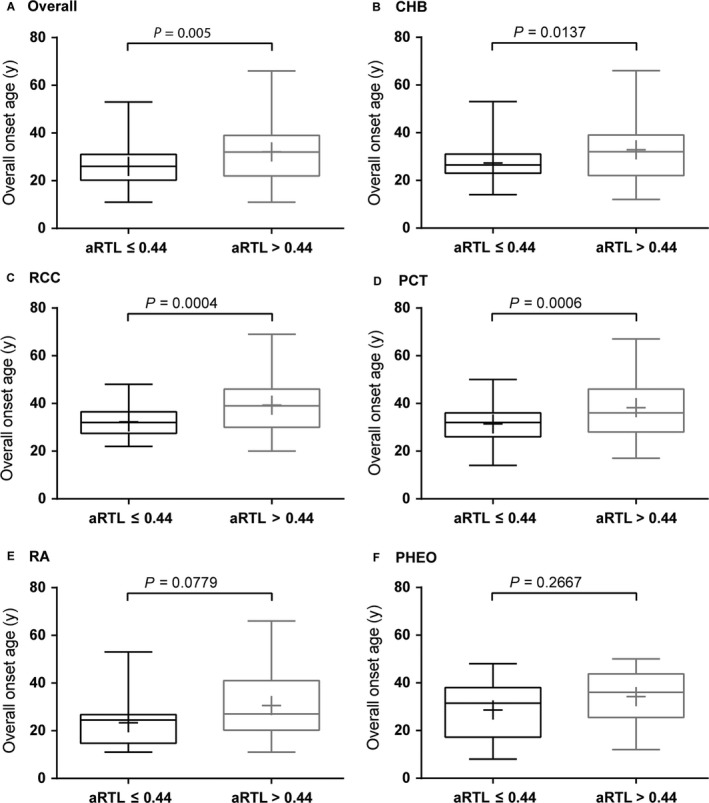
Differences of mean onset age between shorter telomere group and longer telomere group. Patients were divided into shorter telomere group (age‐adjusted RTL ≤ 0.44) and longer telomere group (age‐adjusted RTL > 0.44). Unpaired *t* test with Welch's correction was used to compare the tumor onset age in two groups. The sign “+” in the box represents the mean onset age, and *P* value was showed for overall tumors (A), CHB(B), RCC(C), PCT(D), RA(E) and PHEO(F).

To further support our hypothesis, we divided 184 VHL patients into two groups based on the median aRTL value (0.44). Mean onset age of VHL‐related tumors was calculated from tumor‐affected patients. As expected, the onset age of the VHL‐related tumors was significantly earlier in shorter telomere group than the longer group (Fig. 3A–F). The RA and PHEO subgroups didn't show a significant result due to a limited sample. No difference was found in sex, family history, mutation types and origin (Table 3).

**Table 3:** Genetic and clinical features of the two VHL patient groups

	aRTL ≤ 0.44	aRTL > 0.44	*P* value
	N (%)	N (%)	
Sex			
Male	42 (45.7%)	49 (53.3%)	0.302
Female	50 (54.3%)	43 (46.7%)	
Family history^a^			
Yes	77 (88.5%)	60 (77.9%)	0.068
No	10 (11.5%)	17 (22.1%)	
Mutation			
Missense	50 (54.3%)	43 (46.7%)	0.302
Truncating	42 (45.7%)	49 (53.3%)	
Origin^b^			
Paternal	30 (40.5%)	18 (36.7%)	0.672
Maternal	44 (59.5%)	31 (63.3%)	
Total	92	92	–

aRTL, age‐adjusted relative telomere length.

a 20 patients have an unknown family history.

b 61 patients have an unknown origin.

### Shorter telomere length was associated with higher age‐related tumor risks

**Figure 4:**
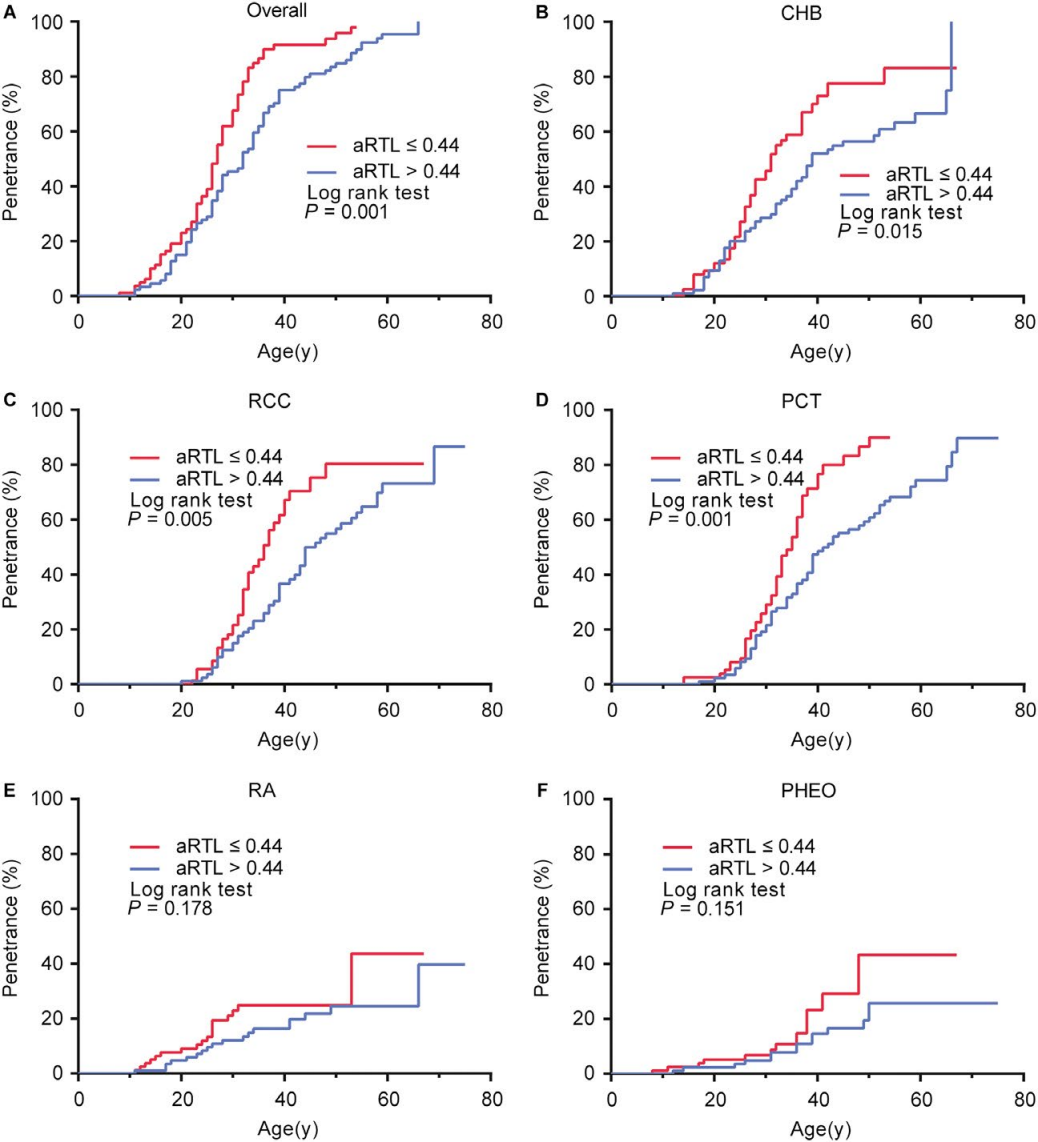
Comparison of age‐related penetrance of VHL‐associated tumors in the shorter telomere group and the longer group. Kaplan–Meier plots describe the distribution of onset age of different tumor types. Log‐rank test was performed to compare the difference between shorter telomere group (red line) and longer telomere group (blue line) in the overall tumors(A), CHB(B), RCC(C), PCT(D), RA(E) and PHEO(F).

We further examined aRTL as a potential predictive factor for age‐related tumor risks in VHL disease. Kaplan–Meier plots and log‐rank analyses were used to describe the distribution of onset age of different tumor types. The distribution of onset age for the first tumor showed an obvious “shift to left” phenomenon in shorter telomere group (*P* = 0.001) (Fig. 4A). The penetrance of overall tumors was higher in the shorter telomere group (67.5% by 30 years and 91.6% by 40 years) than the longer group (45.3% by 30 years and 75.0% by 40 years). Respective analyses for CHB, RCC, PCT, RA and PHEO showed the similar tendency (Fig. 4B–F).

**Figure 5:**
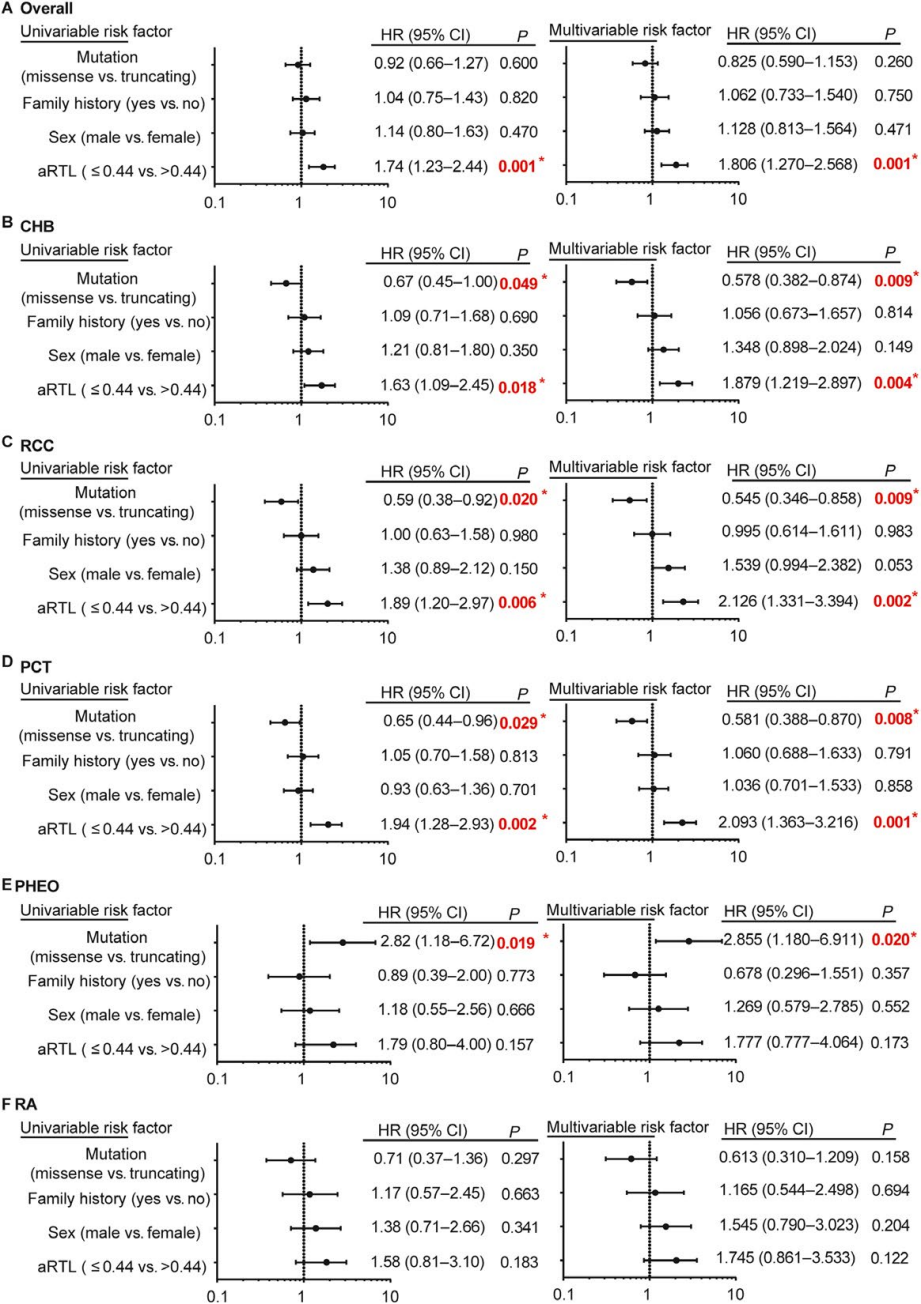
Univariate and multivariate Cox regression analyses for age‐related tumor risks. VHL mutation types (missense vs. truncating), family history, sex and aRTL were brought into the risk factors in the Cox model. HR (95% CI) and *P* value of four risk factors were displayed for overall tumors(A), CHB(B), RCC(C), PCT(D), PHEO (E) and RA (F). The sign “*” represents statistically significant.

Considering the fact that tumor risks for VHL patients may be influenced by sex, family history and mutation types, we performed univariate and multivariate Cox regression analyses to evaluate age‐related tumor risks in VHL patients. The results revealed that patients with shorter telomere had a higher age‐related risks for CHB (HR: 1.88, 95% CI: 1.22–2.90, *P* = 0.004), RCC (HR: 2.13, 95% CI: 1.33–3.39, *P* = 0.002) and PCT (HR: 2.09, 95% CI: 1.36–3.22, *P* = 0.001) (Fig. 5B–D), while there were no significant differences for RA and PHEO which might due to a limited sample (Fig. 5E–F). Meanwhile, we confirmed missense mutation was associated with lower age‐related risks for CHB (HR: 0.60, 95% CI: 0.38–0.87, *P* = 0.009), RCC (HR: 0.55, 95% CI: 0.35–0.86, *P* = 0.009) and PCT (HR: 0.58, 95% CI 0.39–0.87, *P* = 0.008) (Fig. 5B–D), and obviously higher risk for PHEO (HR: 2.86, 95% CI: 1.18–6.91, *P* = 0.020) (Fig. 5E). Altogether, we demonstrated that patients with shorter telomere length were at increased risk for developing VHL–related CHB, RCC and PCT in an early age, despite of the mutation types, sex and family history status.

## Discussion

Hereditary neoplasia syndromes, including VHL disease and Lynch syndrome, usually display a remarkable variability in tumor risks between mutation carriers 30, 31, 32, 33. Although genotype–phenotype correlations have been well described in multiethnic cohorts, the variability cannot be readily explained by mutation types of the related genes. In this study, we show that telomere length is positively correlated with the onset age of five major VHL‐associated tumors in tumor‐affected mutation carriers. Moreover, the shorter telomere group confers an increased age‐related tumor risks for CHB, RCC, and PCT than the longer group, indicating that peripheral blood telomere length may be a tumor risk marker for VHL disease patients.

Comparing the clinical features of VHL disease in UK reported by Ong et al., we found that the first VHL‐associated lesion occurred 6.2 years later in Chinese VHL patients, with surprisingly lower frequencies of CHB and RA. This can be partly explained by the more active molecular genetic testing and widespread surveillance of at‐risk relatives in UK 8. In the UK cohort, 20% patients were diagnosed pre‐symptomatically by molecular genetic analysis, while the proportion was less than 13% in our cohort. However, the frequency of VHL‐related RCC was higher in China, indicating that phenotypic variability may not only exist within and between families, but also between different ethnic groups. Future population‐based studies comparing the VHL genotype, modifier genes, or other factors are required to provide more evidence. The definitely lower frequency of RA in Chinese VHL patients (22.3% vs. 73%) should be taken into consideration when guidelines are made for Chinese patients, and implies some unknown factors participating in the pathogenesis of RA.

Telomere shortening has been proved to be a risk factor in many sporadic and hereditary cancers, including Lynch syndrome, hereditary prostate cancer, familial, and sporadic ovarian cancer. As to RCC, two hospital‐based case–control studies demonstrated that short leukocyte telomere length was associated with increased sporadic RCC risk, while a large population‐based study drew different conclusions 22, 23, 25. Svenson et al. reported that a highly significant association was found between short blood telomeres and a favorable outcome in non‐metastatic RCC patients 34. However, in the hereditary cases, our previous study revealed that VHL patients in the next generation had younger onset age with shorter blood telomere length, while patients in the first generation had older onset age with longer telomere 17. In the current study, we analyzed the data of interfamilial patients, and observed telomere length was positively correlated with the onset age of first lesion of VHL disease, as well as the five major tumors, suggesting that short telomere length may be an additional tumor risk factor for VHL patients. As genotype is definitely correlated to the risks of CHB, RCC, and PHEO, we performed multivariate Cox analysis between two patient groups divided by the median value of aRTL. The results showed the shorter telomere group had a significantly higher age‐related risk for CHB, RCC, and PCT than the longer telomere group. Therefore, shorter telomere is a new independent risk factor for VHL‐associated CHB, RCC, and PCT.

Most of the blood samples in this study were collected after the presence of tumor, raising doubts that the relatively shorter telomere may be the consequence of tumor burden or clinical treatments. To rule out this possibility, we compared the telomere length between tumor‐free mutation carriers and healthy family members, and found that the tumor‐free carriers had an obviously shorter telomere length than the healthy controls (Fig. S1), providing additional evidence that the shorter telomere length was a consequence of VHL gene mutation instead of tumor attack. Further prospective studies assessing telomere length before and after tumor diagnosis in VHL patients will be important to reveal the precise effect of tumor on blood telomere length.

The overexpression of pVHL substrates, like HIF‐1*α* and HIF‐2*α*, plays a critical role in the progression of VHL‐associated tumors. However, the mechanism of tumor initiation in VHL patients remains unclear. As an autosomal dominant syndrome caused by tumor suppressor gene, VHL disease conforms to a classic “two‐hit” model. Loss of heterozygosity (LOH) found in VHL‐related tumor samples verifies the hypothesis. However, how the “second hit” occurs is still unknown. In this study, the blood telomere length in VHL patients was significantly shorter than healthy controls, and patients with shorter telomeres developed tumors in an earlier age. This indicates that shortened telomeres resulting in genomic instability may contribute to the genetic alteration of the wild‐type allele, which accelerates malignant transformation of normal cells. Further basic studies on the mechanisms of telomere shortening and its effect on tumor initiation in VHL patients are needed.

In conclusion, we first investigated the effect of blood telomere length on tumor risks in a large cohort of VHL disease patients. Our findings indicate that shorter telomere length is a new biomarker for tumor risks in VHL patients, which is useful for genetic counseling and prompts future research about the role of telomere shortening in the pathogenesis of VHL‐associated tumors.

## Conflict of Interest

The authors declare no conflict of interest.

## Supporting information


**Figure S1.** Comparison of age‐adjusted relative telomere length between tumor‐free VHL carriers and healthy controls.
