# Supplementary figures and images for: Shorter telomere length increases age‐related tumor risks in von Hippel‐Lindau disease patients
Source: Cancer Med. 2017 Aug 4;6(9):2131–41. doi: 10.1002/cam4.1134 (PMC5603836; doi:10.1002/cam4.1134)

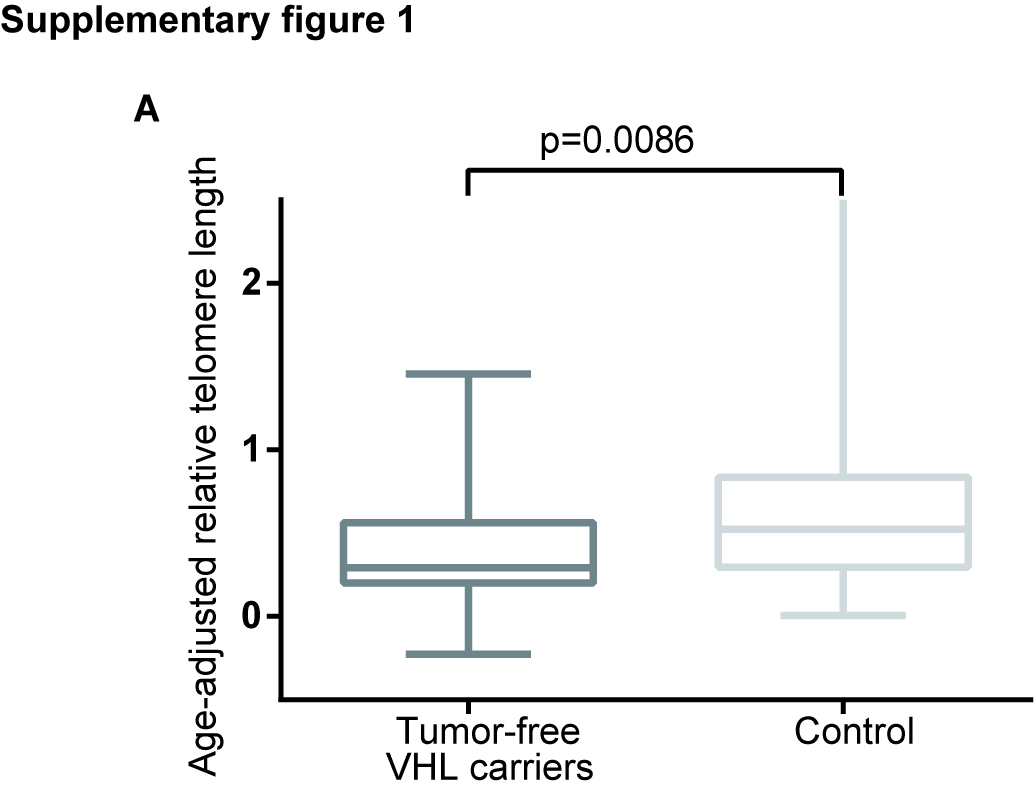

Supplement: Supplementary file 1 — Figure S1. Comparison of age‐adjusted relative telomere length between tumor‐free VHL carriers and healthy controls. [file CAM4-6-2131-s001.tif]
